# Stereotactic Radiosurgery for Recurrent Glioblastoma Multiforme: A Retrospective Multi-Institutional Experience

**DOI:** 10.7759/cureus.18480

**Published:** 2021-10-04

**Authors:** Eduardo E Lovo, Alejandra Moreira, Kaory C Barahona, Juliana Ramirez, Fidel Campos, Carlos Tobar, Victor Caceros, Morena Sallabanda, Kita Sallabanda

**Affiliations:** 1 Radiosurgery/Neurosurgery, International Cancer Center, Diagnostic Hospital, San Salvador, SLV; 2 Neurosurgery, International Cancer Center, Diagnostic Hospital, San Salvador, SLV; 3 Radiation Oncology, International Cancer Center, Diagnostic Hospital, San Salvador, SLV; 4 Radiosurgery, Robotic Radiosurgery Center, San Jose, CRI; 5 Radiosurgery, International Cancer Center, Diagnostic Hospital, San Salvador, SLV; 6 Radiation Oncology, Quirónsalud Proton Therapy Centre, Madrid, ESP; 7 Radiosurgery/Neurosurgery, Hospital Clinico Universitario San Carlos, Madrid, ESP

**Keywords:** recurrence, cyberknife, gamma knife, stereotactic radiosurgery, glioblastoma

## Abstract

Introduction

Glioblastoma multiforme (GBM) is the most common and lethal primary malignancy of the central nervous system. Despite standard therapy protocols, such as aggressive surgical resection, radiotherapy, and chemotherapy, GBM's aggressive nature produces low survival rates. Tumor recurrence and progression are nearly universal. Stereotactic radiosurgery (SRS) has been studied as an alternative treatment for recurrent GBM as a minimally invasive option that might prolong survival. The objective of this retrospective study was to evaluate the efficacy of SRS as a treatment modality considering overall survival (OS) in patients with GBM who had tumor recurrence and were treated with SRS in three different institutions.

Materials and methods

We retrospectively reviewed patients who received SRS for recurrent GBM between 1992 and 2020. A total of 46 patients were included in this study. We recorded age at diagnosis, the extent of surgical resection, radiation treatment, chemotherapy regimen, Karnofsky Performance Status at the time of SRS and at last follow-up, use of adjuvant chemotherapy after SRS, and response evaluation criteria in solid tumors. Primary endpoints were OS after initial diagnosis and OS from the date of the SRS procedure.

Results

Patients received SRS at a median of 10 months (range, 1 to 94 months) after their initial diagnoses. Median follow-up was seven months from the time of SRS and 22.8 months since diagnosis. The estimated median OS for all patients was nine months (range, 1 to 42 months) after SRS and 23.8 months (range, 4 to 102 months) after diagnosis. Median OS after SRS was seven months for patients treated from 1992 to 2011 and nine months for those treated from 2012 to 2020 (p = 0.008; X^2 ^= 7.008). Median OS for younger patients (i.e., those aged <50 years) was 37.1 months compared to 18.6 months for older patients (i.e., those aged >50 years; p = 0.04; X^2 ^= 3.870). Patients who received SRS after 10 months since diagnosis had a median OS of 36.2 months versus those who received SRS sooner than 10 months, who had an OS of 15 months (p = 0.004; X^2 ^= 8.145). Radiosurgery doses larger than 15 Gy correlated with a median survival of nine months versus seven months in those treated with doses <15 Gy (p = 0.01; X^2 ^= 6.756). Lastly, patients who received adjuvant bevacizumab (BEV) and or chemotherapy after SRS had a median survival of 12 months versus seven months for patients who did not receive any additional therapy after SRS (p = 0.04; X^2 ^= 4.196).

Conclusion

SRS focal recurrent GBM in selected patients may improve OS, especially when combined with adjuvant therapy such as BEV and chemotherapy. Other prognostic variables proved relevant such as patients' age, the dose delivered, and surgery-to-SRS time that translates to the time of recurrence. Our results were consistent with the published literature and added to the accumulating evidence regarding SRS in recurrent GBM; however, extensive, multi-center studies are required to make definitive recommendations on this treatment approach.

## Introduction

Glioblastoma multiforme (GBM) is the most common and deadliest primary malignancy of the central nervous system [1]. Because of its aggressive and infiltrative nature and the complexity of its pathophysiology, GBM is one of the most challenging tumors to treat [2]. Almost universally, this tumor recurs with progression-free survival (PFS) typically less than nine months and survival that hovers at 12 to 16 months, regardless of the multimodality treatment strategies that can include aggressive surgical resection, chemotherapy, and radiotherapy [3]. Only a few patients survive 2.5 years, and fewer than 5% of patients survive five years following the original diagnosis [4,5]. Different treatment modalities for tumor recurrence include surgery, radiosurgery, targeted immunotherapy, and tumor treating fields. Nevertheless, recurrence has nearly universal mortality and median survival after recurrence between 9 and 20 months [6-8], depending on the treatment strategy.

Currently, no standard of care recommendation is established for recurrent GBM, and the approach can be heterogeneous. Bevacizumab (BEV), a humanized monoclonal antibody against vascular endothelial growth factor (VEGF), was introduced to standard treatment with temozolomide, and although it did not show to increase overall survival (OS), it improved PFS [7,9]. Resection of recurrent GBM is an alternative, but surgery alone is insufficient for disease control due to the infiltrative nature of the disease. Also, GBM often involves additional areas in the patient's brain that deteriorate the standard baseline neurological or physiological condition, limiting surgical access [10]. Conventional or hypofractionated radiotherapy reirradiation as treatment modalities have a median OS ranging from six to nine months, but potential accumulative toxicity and the risk for radiation necrosis limits the regular applicability of those treatments [11].

Stereotactic radiosurgery (SRS) has been studied as a treatment modality for recurrent GBM for more than two decades. To date, only one prospective randomized trial has been published investigating the effect of SRS added to conventional external beam radiation therapy on the survival of patients with newly diagnosed GBM, and the authors found no benefit to patients when giving SRS as a boost before standard radiotherapy and carmustine (BCNU) [12]. Evidence regarding SRS in tumor recurrence is inconclusive for establishing SRS as standard practice [6,13-16]. SRS seems like an attractive alternative due to its minimally invasive nature for focal recurrences. Further, its submillimeter accuracy and steep dose gradient seem reasonable for recurrence as patients have previously received a high dose of radiation, and few other noninvasive alternatives such as tumor-treating fields (TTF) are not widely available.

We conducted a retrospective study in three different radiosurgery centers of patients with GBM who were treated at the time of tumor recurrence with SRS to evaluate the efficacy of SRS as a treatment modality. We evaluated treatment outcome and OS.

## Materials and methods

We retrospectively reviewed patients who received SRS for recurrent glioblastoma at three radiosurgery centers between January 1992 and December 2020, and a total of 46 patients were included in this study. This study was approved by the ethics committees of the three centers (International Cancer Center of the Diagnostic Hospital, the Robotic Radiosurgery Center, and Instituto Madrileno de Oncologia [IMO]), and all patients provided informed consent for their treatment. One center used the frame-based rigid fixation SRS system 200 developed by the Gainesville University of Florida in a Precise Linear Accelerator (LINAC) developed by Elekta (Stockholm, Sweden). The second center used CyberKnife (Accuray, Sunnyvale, California, USA), and the third center used Infini gamma-ray (GR), rotating, intracranial, SRS system (Masep Medical Company, Shenzhen, China).

We evaluated age at diagnosis, the extent of surgical resection, radiation treatment modality, chemotherapy, and radiographic evidence of tumor recurrence/progression versus tumor necrosis (defined by a contrast-enhanced and perfusion-weighted magnetic resonance imaging [MRI] when available), and time to tumor progression. Karnofsky Performance Status (KPS) at the time of SRS and last follow-up, adjuvant chemotherapy after SRS, and response evaluation criteria in solid tumors (RECIST) were also reviewed. The primary endpoint was OS after the initial diagnosis of glioblastoma and OS from the date of the SRS procedure. To calculate OS, patients were contacted via phone call to estimate the last day of follow-up if they were alive, and if patients could not be identified, death dates verified with family members were considered.

Radiosurgical technique

Of the patients included, 24 were treated with a cone-based radiosurgery system. A rigid fixation frame was placed under local anesthetic by a neurosurgeon, computed tomography (CT) was acquired and fused with a T1 gadolinium 1.2-mm MRI, and treatment planning was done in accordance by radiation oncology and neurosurgery with the treatment planning station LINAC Scalpel and Radionics X-Knife Software (Integra LifeScience Holding Corporation, Massachusetts, USA). After treatment, the head frame was removed, and patients were discharged home.

For the 18 patients treated with Infini, we obtained a volumetric MRI with stereotactic frame rigidity fixed to the head under local anesthesia, a T1-weighted multiplanar gradient recall gadolinium with 1-mm thickness slices in the axial orientation, as well as a perfusion sequence map. Treatment plans were prepared jointly by a neurosurgeon and a radiation oncologist with target volumes based on the T1 postcontrast sequence and areas suggested by perfusion-weighted images to uphold most of the viable tumor mass. Typically, 1-mm tumor margin was included in most treatment plans using the Superplan (Masep Medical Company, Shenzhen, China) treatment planning station.

Patients who underwent SRS with CyberKnife (CK) received a CT simulation three to five days before the treatment to assess the tumor's size, location, and shape and create a thermoplastic mask for immobilization. We obtained 1.25-mm slice CT images in combination with T1 gadolinium with 1-mm slices in the axial orientation and perfusion sequence map (when available). Treatment fractions ranged from one to five days.

Statistical analysis

We applied the Shapiro-Wilk test to determine the normal distribution of values. Based on value distribution, measures of central tendency were used for categorical and continuous variables. Median OS was estimated with the Kaplan-Meier survival analysis, and the set of covariates selected to assess their impact on OS consisted of age, sex, radiosurgical technique, use of adjuvant chemotherapy, dose, volume, the time between diagnosis, and SRS. P-values <0.05 indicate statistical significance.

## Results

Patient demographics and treatment history before SRS are shown in Table 1.

**Table 1:** Patient demographic and clinical criteria. Abbreviations: BCNU, carmustine; SRS, stereotactic radiosurgery; BED, biological effective dose; BEV, bevacizumab.

Data category	N
Patients (n)	46
Male (%)	28 (60.9%)
Female (%)	18 (39.1%)
Mean age in years (range)	50.3 (19-81)
Extent of first surgical resection	
Complete resection (%)	20 (43.5%)
Partial resection (%)	23 (52.2%)
Biopsy (%)	3 (4.3%)
Radiotherapy post-surgery	46 (100%)
Chemotherapy regimen	
Temozolomide (%)	16 (34%)
Temozolomide + Bevacizumab (%)	6 (13%)
BCNU (%)	24 (52%)
Median time between initial diagnosis, recurrence, and SRS in months (range)	10 (1-94)
Median prescription dose (Gy) calculated by BED to a single fraction when multiple fractions were delivered using an alpha/beta of 10	
Cone-based SRS, single fraction (range)	14 (8-20)
Infini, single fraction (range)	15 (10-24)
CyberKnife, single/multiple fraction SRS (range)	16 (15-17)
Median tumor volume	
Cone-based SRS	5.6cc (0.2-65.5 cc)
Infini	3.2cc (0.1-64.8 cc)
CyberKnife	36.9cc (6-71 cc)
Concurrent/adjuvant BEV and or chemotherapy with SRS	
Received concurrent or adjuvant chemotherapy (%)	15 (32.6%)
Did not receive concurrent or adjuvant chemotherapy (%)	31 (67.3%)

We analyzed 46 patients, of whom 28 (60.9%) were men and 18 (39.1%) were women. The mean age at SRS was 50.3 years (range, 19 to 81 years). Complete surgical resection of the tumor at first diagnosis was documented in 20 patients (43.5%); 23 (52.2%) patients had partial tumor resection, and three (4.3%) patients had biopsy only. All patients underwent adjuvant radiotherapy and chemotherapy after surgery; 24 (52%) patients received BCNU, 16 (34%) patients received temozolomide alone, and six (14%) patients received temozolomide and BEV.

Time to recurrence and treatment

Patients received SRS at a median of 10 months (range, 1 to 94 months) after initial diagnosis. Single lesions were documented and treated in 35 (76%) patients, and 11 (23.9%) patients had multiple lesions treated at the time of SRS. A group of five (10.9%) patients received a second SRS treatment session due to tumor recurrence, four (80%) from GR, and one (20%) from CK; median duration between the first and second treatment was 4.5 months (range, 1 to 44 months). The median volume for cone-based SRS was 5.4 cc (range, 0.2 to 65.5 cc), and the median prescription dose was 14 Gy (range, 8 to 20 Gy). Median GR treatment volume was 3.2 (0.1-64.8), and the median prescription dose was 15 Gy (10 to 24 Gy) considering single and multiple targets. Median CK treatment volume was 36.9 cc (range, 6 to 71 cc), and the median prescription dose was 16 Gy (range, 15 to 17 Gy). Two treatments were delivered in a single fraction, and two were delivered in multiple (five) fractions; the prescription dose was calculated in equivalence to biological effective dose to a single fraction with an alpha/beta of 10.

We also reviewed the use of adjuvant/concurrent BEV and chemotherapy with SRS treatment. Only 15 (32.6%) patients received concurrent or adjuvant chemotherapy, 12 (80%) received BEV, and three (20%) had temozolomide alone. KPS was considered at the time of SRS, and 37 (80.4%) patients were able to carry out everyday activities without special care (KPS of 90; range, 80 to 100), and nine (19.6%) were unable to work but able to live at home and care for personal needs while requiring varying amounts of assistance (KPS of 60; range, 50 to 70).

SRS post-treatment outcomes

Median follow-up was seven months from the time of SRS and 22.8 months since diagnosis. The estimated median OS for all patients was nine months (range, 1 to 42 months) after SRS and 23.8 months (range, 4 to 102 months) after diagnosis. Median OS after SRS was seven months for patients treated from 1992 to 2011 and 25.7 months since diagnosis. Median OS after SRS was nine months and 20 months after diagnosis for patients treated from 2012 to 2020. Regarding RECIST, 12 (54%) patients were alive of those treated during 2012 to 2020, two (16.6%) reported having tumor progression documented on MRI, six (50%) reported having local control on MRI, and four (33.3%) patients did not have a control MRI at last follow-up.

The Kaplan-Meier estimator was used to measure the fraction of patients living for a certain amount of time after receiving SRS. Estimations were also made from the time of diagnosis to determine the OS.

**Figure 1:**
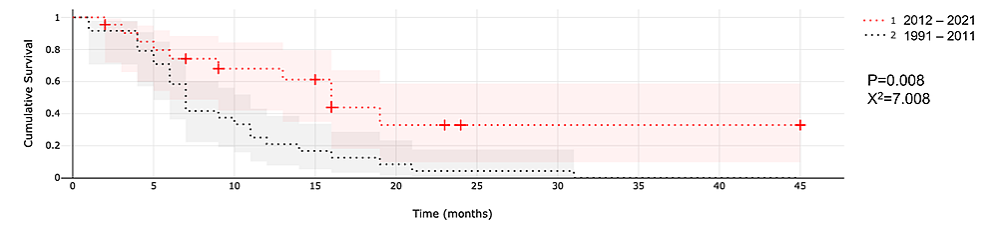
Comparison of median survival after SRS between different time frames. Abbreviation: SRS, stereotactic radiosurgery.

Median survival after SRS was compared between both time frames (1992 to 2011 and 2012 to 2020). Median OS was nine months and 20 months since diagnosis for patients treated during the second period (2012 to 2020). We noted a two-month difference of median survival after SRS was found between time frames (p = 0.008, X^2^ = 7.008). Median OS since the time of diagnosis was not statistically significant (25.7 versus 20 months; p = 0.947; X^2 ^= 0.004) between both periods (Figure 1).

**Figure 2:**
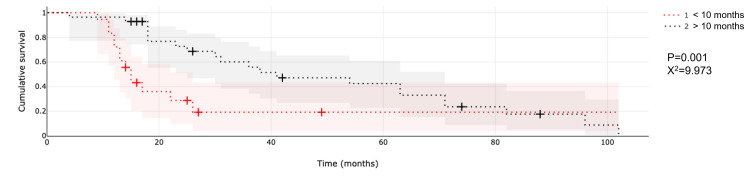
Comparison of overall survival between patients who received SRS prior to or after 10 months of diagnosis. Abbreviation: SRS, stereotactic radiosurgery.

An analysis of the time of surgery to recurrence and, thus, SRS treatment time revealed that a longer interval (>10 months) between diagnosis and time of SRS correlates with longer OS. Patients who received SRS after 10 months since diagnosis had a median OS of 36.2 months, while those who received SRS sooner than 10 months from diagnosis had an OS of 15 months (p = 0.004; X^2^ = 8.145; Figure 2).

**Figure 3:**
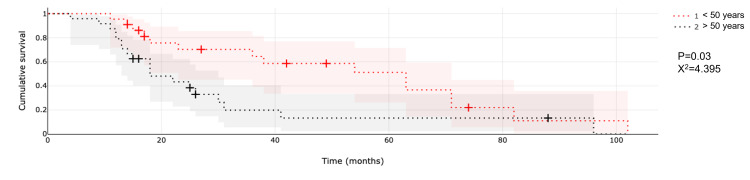
Comparison of median survival between ages.

Patient age was a significant factor in median OS (Figure 3).

Median OS since diagnosis for younger patients (age <50 years) was 37.1 months vs. 18.6 months for older patients (age >50 years; p = 0.04; X^2 ^= 3.870). Although patient age was not significant in the median survival from the time of SRS (p = 0.09), median survival for younger patients is 11.5 months compared to six months for older patients; a six-month difference of median survival is worth mentioning given the prognosis of the disease.

**Figure 4:**
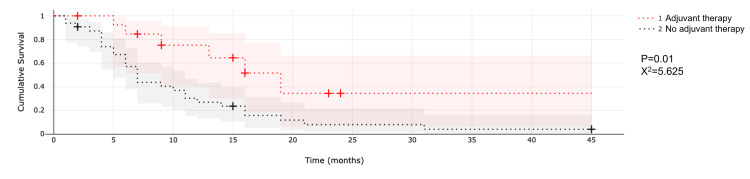
Comparison of median survival after SRS between patients who received or did not receive adjuvant therapy. Abbreviation: SRS, stereotactic radiosurgery.

Another variable considered was the use of adjuvant/concomitant BEV and/or chemotherapy after SRS (Figure 4).

Patients who received adjuvant treatment to SRS with either BEV or temozolomide had a median survival of 12 months compared to a median survival of seven months for patients who did not receive treatment after SRS (p = 0.04; X^2^ = 4.196).

**Figure 5:**
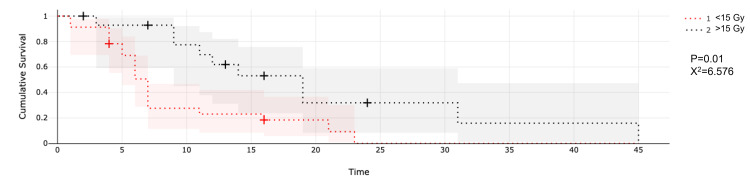
Comparison of single-session radiosurgery with doses larger or lower than 15 Gy.

Patients treated with a prescription dose larger than 15 Gy showed a median survival of nine months compared to seven months in those treated with <15 Gy (p = 0.01; X^2^ = 6.756; Figure 5).

Median tumor volume was 2.9 cc, 36.85 cc, and 5.4 cc in GR, CK, and cone-beam SRS, respectively. Tumor volume did not impact the median survival (p = 0.494, p = 0.08, and p = 0.622, respectively).

## Discussion

The prognosis of GBM, despite multimodal therapies including surgery, radiation, and chemotherapy, remains grim. The disease is characterized by aggressive local invasion, with recurrences developing proximal to the tumor's original site in most cases. Therefore, approaches like surgery, SRS alone, or SRS with additional treatment options have been explored, producing different survival rates. Independent predictors of survival in patients with GBM treated with SRS have been evaluated to determine their influence in the median survival, including dose, time of SRS from the time of diagnosis, and age.

The current study analyzed the survival rate of patients with glioblastoma treated with SRS in three different centers over nearly 30 years. Patients treated from 1992 to 2011 had a median survival of seven months, whereas the median survival for patients treated from 2012 to 2020 had a more prolonged survival of nine months (p = 0.02). Median OS seemed higher in patients treated in the earlier time frame, although the difference was not statistically significant (35.3 vs. 29.5 months; p = 0.415). Making such a comparison between timeframes allowed us to compare survival outcomes between patients who received SRS with concomitant chemotherapy compared to SRS alone. It is worth mentioning that all patients treated during the first interval did not receive adjuvant chemotherapy because it was not an approved therapeutic option, nor were current regimens available.

Some series that have evaluated patients who received BEV along with SRS and compared them to those who received SRS alone found that patients who received BEV had longer rates of PFS and OS (median PFS was 5.2 vs. 2.1 months; median OS was 11.2 vs. 3.9 months) [16,17]. Also, adjuvant bevacizumab to reirradiation for recurrent glioblastoma showed a median PFS of 7.3 months and an OS of 12.4 months in 25 patients [17]. A study of dose escalation of single-fraction radiosurgery for recurrent glioblastoma in the setting of BEV therapy found that pre-SRS BEV treatment (10 to 14 days before SRS) was associated with a reduction of the mean volume of the enhancing lesion from 4.7 to 2.86 cm^3^. However, this was not a statistically significant reduction (p = 0.103). Median PFS and OS were 7.5 and 12 months, respectively [2].

Within our study, patients who received mainly adjuvant treatment to SRS with BEV and, to a lesser extent, temozolomide had a median survival of 12 months compared to those who did not receive any form of adjuvant therapy who had a median survival of seven months (p = 0.04).

Bevacizumab, as an anti-VEGF, has been shown to decrease interstitial fluid pressure and normalize the tumor vasculature. It also decreases edema and prevents radionecrosis after SRS [18-21]. Pairing SRS with BEV has a double effect on the endothelial cells: SRS is cytotoxic to vascular cells, high-dose radiation causes microvascular endothelial apoptosis, and BEV sensitizes endothelial cells to high-dose radiation [22]. Additionally, high-dose SRS has a vascular endothelial cell ablative effect that is more potent than that observed in radiotherapy [23].

The time between initial surgery and SRS/recurrence was considered an independent factor associated with OS, and we noted a significant association between the surgery to SRS and post-SRS OS; patients with the most extended intervals (>20.2 months) had improved survival (median OS: 15.1 months; p = 0.001) compared to those treated between 15 and 20 months after surgery (median OS: 8.3 months) [6]. In the current series, we also found that a longer interval (>10 months) between diagnosis and time of SRS correlates with longer OS. Patients who received SRS after 10 months since diagnosis had a median OS of 36.2 months, while those who received SRS in under 10 months since diagnosis had an OS of 15 months (p = 0.004). The time interval between diagnosis and SRS did not contribute to median survival since SRS (p = 0.364). Other authors have reported that the interval from primary diagnosis to SRS did not correlate with OS to SRS [16]. In agreement with Imber et al. [6], we believe the patients with a longer recurrence time have a slower progression and, thus, a slightly more indolent disease. Contrarily, patients treated for recurrence sooner than 10 months may have a more aggressive tumor and faster-growing recurrences. No significant differences in demographics, type of microsurgical resection, or radiation treatment modality seemed to explain such findings. A lack of in-depth molecular analyses of the tumors was a limitation in our study.

Glioblastoma is more commonly diagnosed between the ages of 55 and 85, and in the USA, the median age of presentation is 64 years. The patients' ages and medical comorbidities might predict the survival of patients with GBM and their likelihood of receiving and withstanding aggressive treatment. Some authors have reported age as an important prognostic variable, reporting that younger patients (<60 years) at the time of diagnosis had a longer survival time [24]. Other studies reported that patients younger than 50 years at the time of radiosurgery had improved OS from SRS [6,16]. We found that patient age influenced the median OS: median OS since diagnosis for younger patients (age <50 years) was 37.1 months compared to 18.6 months for older patients (age >50 years; p = 0.04). Although patient age was not significant in the median survival from the time of SRS, a six-month difference of median survival among our results is remarkable considering the prognosis of the disease. Our results were in accordance with the additional published literature [25-29]. Despite considering survival since the time of diagnosis or SRS, most authors have found that younger ages correlated with improved survival. Younger patients may have better tolerance to procedures and, presumably, better prognosis. A patient's performance status may be a better predictor of treatment tolerability and survival than the age at diagnosis [9], which can also be considered among younger populations. Nevertheless, no significant association was found among our patients when considering the KPS at SRS time (p = 0.165).

Radiation dose is selected based on several considerations, including the patient's performance status, tumor volume, prior radiation doses to the area, and proximity to eloquent areas. A meta-analysis found that the most used median prescription was 16 Gy [10]. Other authors have also reported that the use of marginal radiation doses greater than 15 Gy is an important prognostic factor to patient survival [13]. In a different report, the same authors found that median survival for the patient who received margin doses of 15 Gy was 12 months compared to 8.2 months for those who received less than 15 Gy [24]. The current study also demonstrated that patients treated with doses larger than 15 Gy had a median survival of nine months versus seven months in those treated with <15 Gy (p = 0.01), similar to that reported in the literature.

Median tumor volume was 2.9 cc, 5.4 cc, and 36.85 cc with GR, cone-based, and CK SRS, respectively. Tumor volume in our series did not impact the median survival since SRS (p = 0.494, p = 0.08, and p = 0.622, respectively). Concurrent with our study findings, similar studies found that treatment volume was not significantly correlated with survival or progression [6,16]. Nevertheless, several studies have shown that tumor volume is a significant prognostic factor and suggested a series of cut-off values as surrogates of survival prognosis, suggesting that low-volume tumors are more suitable for SRS [13,15,29]. A study considering 297 patients reported that smaller tumors (<14 cm^3^) are associated with better OS [13].

Limitations

There are some limitations to consider in this retrospective study. There were no strict patient selection criteria-patients diagnosed with recurrent GBM was the only filter. The population in the study is heterogeneous-some patients had different therapy protocols and different chemotherapeutic regimens due to the wide interval encompassed by this study. Although the study aimed to determine the effectiveness of SRS in the recurrence of GBM, additional therapies that have also contributed to the increase in survival rate should be considered. Our study is generalizable because the subgroups presented portray a direct comparison between treatment protocols and their benefits and a teaching path to which prognostic factors can be considered when treating recurrent GBM with radiosurgery.

## Conclusions

SRS may benefit focal recurrences for patients with recurrent GBM who have previously received high-dose radiation therapies. Based on the results presented herein, GBM patients receiving SRS at the time of recurrence along with concomitant BEV and or chemotherapy can anticipate a superior survival rate compared to what has been reported with single therapeutic modalities. There are essential aspects to consider when treating GBM with SRS, such as patient age, dose delivered, surgery-to-SRS time, and concomitant therapy. Our results were consistent with the published literature and further support the existing evidence; nevertheless, additional multi-institutional studies are required to make definitive recommendations on this treatment modality.
